# Supplementary material for: Marine reserve benefits and recreational fishing yields: The winners and the losers
Source: PLoS One. 2020 Dec 10;15(12):e0237685. doi: 10.1371/journal.pone.0237685 (PMC7728224; doi:10.1371/journal.pone.0237685)
Supplement: S1 Table — (PDF) [file pone.0237685.s001.pdf]

S1 Table List of the species recorded for each of the three major fish families caught by recreational fishermen around the Cerbère-Banyuls natural marine reserve (see Fig 1). Trophic level and maximum length (in cm) from www.fishbase.org.

Family	*Sp.* latin name	*Sp.* common name	Trophic level	Max. length
Sparidae	*Boops boops*	bogue	2.8	40
	Dentex dentex	common dentex	4.5	100
	Diplodus annularis	annular seabream	3.6	28
	Diplodus cervinus	zebra seabream	3.0	55
	Diplodus puntazzo	sheephead bream	3.2	60
	Diplodus sargus	white seabream	3.4	45
	Diplodus vulgaris	two-banded seabream	3.5	45
	Lithognathus mormyrus	sand steenbras	3.4	55
	Oblada melanura	saddled seabream	3.4	37
	Pagellus acarne	axillary seabream	3.8	36
	Pagellus bogaraveo	blackspot seabream	4.2	70
	Pagellus erythrinus	common pandora	3.5	60
	Pagrus pagrus	common seabream	3.9	91
	Sarpa salpa	cow bream	2.0	51
	Sparus aurata	gilt-head bream	3.7	70
	Spondyliosoma cantharus	black seabream	3.3	60
Serranidae	*Anthias anthias*	swallowtail seaperch	3.8	27
	Serranus cabrilla	comber	3.4	40
	Serranus scriba	painted comber	3.8	36
Labridae	*Coris julis*	Mediterranean rainbow wrasse	3.4	30
	Labrus merula	brown wrasse	3.6	45
	Labrus mixtus	cuckoo wrasse	3.9	40
	Labrus viridis	green wrasse	3.9	47
	Symphodus ocellatus	ocellated wrasse	3.5	12
	Symphodus tinca	East Atlantic peacock wrasse	3.3	44
