# Supplementary material for: Marine reserve benefits and recreational fishing yields: The winners and the losers
Source: PLoS One. 2020 Dec 10;15(12):e0237685. doi: 10.1371/journal.pone.0237685 (PMC7728224; doi:10.1371/journal.pone.0237685)
Supplement: S2 Table — (PDF) [file pone.0237685.s002.pdf]

S2 Table Deviance table of generalized linear models characterizing changes in catch per unit effort (CPUE) and weight per unit effort (WPUE) of recreational fishermen fishing on-shore from the coastline versus off-shore from boats, and inside versus outside the Cerbère-Banyuls marine reserve. Separate two-factor models (referred to as model sets) characterized CPUE and WPUE trajectories as a function of *Time*, *Reserve*, and their interaction (*Time* × *Reserve*). Changes in fishing yields are characterized for all species combined (model sets 1-3) as well as separately for each of the three major fish families captured by recreational fishermen: Sparidae, Serranidae, and Labridae (model sets 4-9). For Sparidae, for which yields were increasing, an additional model was used to compare WPUE trajectories between on- versus off-shore, indistinctly from fishermen location inside or outside of the reserve (model 10). The corresponding trajectories are illustrated in Figs. 2 (model set 1), 3 (model sets 2 and 3), and 4 (model sets 4-9).

				**CPUE**		**WPUE**		
**Species**	**Shore**	**Reserve**	**Factor**	**deviance**	***p*-value**	**deviance**	***p*-value**	**Model set**
all	both	in vs out	*Time*	50.26	0.0000	1.89	0.1687	1
			Reserve	3.84	0.0499	6.02	0.0141	
			Time × *Reserve*	0.41	0.5244	10.81	0.0010	
	on	in vs out	*Time*	2.84	0.0919	5.32	0.0210	2
			Reserve	3.35	0.0670	1.82	0.1772	
			Time × *Reserve*	0.12	0.7319	1.32	0.2509	
	off	in vs out	*Time*	38.42	0.0000	0.17	0.6799	3
			Reserve	2.21	0.1372	3.19	0.0741	
			Time × *Reserve*	2.64	0.1042	1.74	0.1868	
Sparidae	on	in vs out	*Time*	0.9080	0.3407	4.9491	0.0261	4
			Reserve	1.4254	0.2325	1.1525	0.2830	
			Time × *Reserve*	0.5519	0.4575	3.2102	0.0732	
	off	in vs out	*Time*	0.4075	0.5232	3.82	0.0505	5
			Reserve	1.6883	0.1938	0.6265	0.4287	
			Time × *Reserve*	0.7246	0.3946	0.37	0.5424	
	on vs off	both	*Time*			0.07	0.7983	10
			Fishermen			7.64	0.0057	
			Time × *Fishermen*			10.32	0.0013	
Serranidae	on	in vs out	*Time*	1.87	0.1720	0.07	0.7891	6
			Reserve	3.61	0.0576	0.9762	0.3231	
			Time × *Reserve*	4.07	0.0436	0.1281	0.7204	
	off	in vs out	*Time*	36.75	0.0000	10.33	0.0013	7
			Reserve	12.96	0.0003	11.16	0.0008	
			Time × *Reserve*	0.17	0.6765	0.01	0.9076	
Labridae	on	in vs out	*Time*	3.81	0.0510	7.40	0.0065	8
			Reserve	0.54	0.4613	0.23	0.6316	
			Time × *Reserve*	0.01	0.9134	0.31	0.5791	
	off	in vs out	*Time*	4.19	0.0407	2.07	0.1506	9
			Reserve	7.81	0.0052	0.40	0.5261	
			Time × *Reserve*	3.45	0.0632	0.00	0.9596	
