# Supplementary material for: Marine reserve benefits and recreational fishing yields: The winners and the losers
Source: PLoS One. 2020 Dec 10;15(12):e0237685. doi: 10.1371/journal.pone.0237685 (PMC7728224; doi:10.1371/journal.pone.0237685)
Supplement: S3 Table — (PDF) [file pone.0237685.s003.pdf]

S3 Table Deviance table of generalized linear models comparing average catch per unit effort (CPUE) and weight per unit effort (WPUE) of recreational fishermen fishing on-shore from the coastline and off-shore from boats, and inside versus outside the Cerbère-Banyuls marine reserve over the entire study period (averaged over 2005-2014). Separate single-factor models were used to compare average CPUE and WPUE values between fishing zones (inside versus outside the reserve) and fishermen groups (on- versus off-shore).

				**CPUE**		**WPUE**	
**Species**	**Shore**	**Reserve**	**Factor**	**deviance**	***p*-value**	**deviance**	***p*-value**
all	both	in vs out	*Reserve*	9.22	0.0024	5.61	0.0178
	on vs off	both	*Fishermen*	171.10	0.0000	73.99	0.0000
Sparidae	both	in vs out	*Reserve*	0.65	0.4217	0.22	0.6413
	on vs off	both	*Fishermen*	25.83	0.0000	7.46	0.0063
Serranidae	both	in vs out	*Reserve*	23.12	0.0000	15.63	0.0000
	on vs off	both	*Fishermen*	215.96	0.0000	105.92	0.0000
Labridae	both	in vs out	*Reserve*	3.59	0.0583	0.25	0.6155
	on vs off	both	*Fishermen*	0.11	0.7417	0.15	0.6958
