# Supplementary material for: Marine reserve benefits and recreational fishing yields: The winners and the losers
Source: PLoS One. 2020 Dec 10;15(12):e0237685. doi: 10.1371/journal.pone.0237685 (PMC7728224; doi:10.1371/journal.pone.0237685)
Supplement: S4 Table — (PDF) [file pone.0237685.s004.pdf]

S4 Table Fishing yields (mean ±95% confidence interval) in catch per unit effort (CPUE in numbers per line per hour) and weight per unit effort (WPUE in grams per line per hour) estimated for recreational fishermen performing on- and off-shore, inside and outside of the Cerbère-Banyuls marine reserve at the beginning (*i*) and end (*f*) of the survey (2005-2014). Ratios (R*f*/*i*) indicate the proportional change in yields between the two dates (e.g. 0.50 indicates a decline with -50% in yield, and 2.00 indicates an increase with +100% in yield). Values are provided for all species combined as well as separately for each of the three major fish families captured by recreational fishermen (Sparidae, Serranidae, Labridae). The corresponding trajectories are illustrated in Figs. 2-4.

**Sp.**	**Shore**	**Reserve**	**CPUE*i***	**CI*i***	**CPUE*f***	**CI*f***	**R*f*/*i***	**WPUE*i***	**CI*i***	**WPUE*f***	**CI*f***	**R*f*/*i***
all	both	in	2.54	2.06-3.14	1.07	0.71-1.60	0.42	222.25	157.81-313.01	514.01	267.30-988.44	2.31
		out	2.22	1.85-2.67	0.76	0.59-0.97	0.34	275.25	204.90-369.76	110.13	74.15-163.58	0.40
all	on	in	0.60	0.43-0.85	0.37	0.15-0.89	0.61	74.33	34.34-160.86	52.79	7.08-393.41	0.71
		out	1.02	0.75-1.39	0.47	0.34-0.67	0.47	202.18	98.62-414.52	29.53	13.52-64.50	0.15
	off	in	4.00	3.11-5.15	1.05	0.68-1.61	0.26	336.73	241.78-468.97	543.40	312.74-944.19	1.61
		out	2.89	2.34-3.57	1.29	0.92-1.81	0.45	320.71	244.12-421.34	264.65	169.30-413.70	0.83
Spar	on	in	0.29	0.18-0.48	0.08	0.02-0.30	0.27	34.18	14.63-79.87	68.04	7.53-614.76	1.99
		out	0.36	0.23-0.56	0.24	0.15-0.39	0.66	177.41	80.53-390.84	12.75	5.41-30.03	0.07
	off	in	0.52	0.36-0.76	0.45	0.24-0.83	0.86	107.35	52.67-218.79	191.31	58.17-629.22	1.78
		out	0.55	0.40-0.75	0.74	0.46-1.19	1.35	70.77	39.30-127.44	229.48	87.90-599.08	3.24
Serr	on	in	0.02	0.01-0.06	0.10	0.02-0.52	4.04	2.03	0.84-4.88	2.38	0.24-23.48	1.17
		out	0.20	0.11-0.35	0.03	0.01-0.06	0.14	6.56	2.89-14.89	3.22	1.32-7.83	0.49
	off	in	3.17	2.11-4.75	0.48	0.24-0.97	0.15	226.54	108.21-474.28	48.99	14.29-168.04	0.22
		out	1.54	1.09-2.2	0.30	0.17-0.52	0.19	88.43	48.15-162.39	21.18	7.92-56.66	0.24
Labr	on	in	0.27	0.11-0.69	0.10	0.01-1.12	0.35	21.74	5.08-93.05	1.55	0.04-69.73	0.07
		out	0.37	0.16-0.88	0.12	0.05-0.32	0.33	12.45	3.19-48.87	2.48	0.56-10.89	0.20
	off	in	0.26	0.11-0.57	0.00	0.00-0.02	0.02	9.46	2.76-32.40	1.63	0.21-12.83	0.17
		out	0.48	0.26-0.91	0.15	0.05-0.41	0.30	14.15	5.13-39.05	2.71	0.53-13.98	0.19
