# Supplementary material for: Marine reserve benefits and recreational fishing yields: The winners and the losers
Source: PLoS One. 2020 Dec 10;15(12):e0237685. doi: 10.1371/journal.pone.0237685 (PMC7728224; doi:10.1371/journal.pone.0237685)
Supplement: S5 Table — (PDF) [file pone.0237685.s005.pdf]

S5 Table Parameter estimates (in log link scale) of generalized linear models characterizing trends in catch per unit effort (CPUE) and weight per unit effort (WPUE) of recreational fishermen fishing on-shore from the coastline versus off-shore from boats, and inside versus outside the Cerbère-Banyuls marine reserve. Values are provided for all species combined as well as separately for each of the three major fish families captured by recreational fishermen (Sparidae, Serranidae, Labridae). SE indicates standard error, and *p*-value indicates whether the estimated intercept and slope parameters differ significantly from zero. The corresponding trajectories are illustrated in Figs. 2-4.

Species	Shore	Reserve	Metric	Intercept	SE	*p*-value	Slope	SE	*p*-value
All	both	in	CPUE	4.1779	1.0611	0.0001	-0.0003	0.0001	0.0010
		out	CPUE	4.8277	0.7805	0.0000	-0.0001	0.0001	0.5181
		in	WPUE	2.2447	1.7200	0.1919	0.0002	0.0001	0.0481
		out	WPUE	9.0455	1.2415	0.0000	-0.0005	0.0002	0.0007
	on	in	CPUE	1.3808	2.6981	0.6088	-0.0001	0.0002	0.4680
		out	CPUE	2.7740	1.5966	0.0823	-0.0001	0.0002	0.7613
		in	WPUE	5.6188	4.7856	0.2404	-0.0001	0.0004	0.7736
		out	WPUE	12.5136	2.8059	0.0000	-0.0005	0.0004	0.2562
	off	in	CPUE	6.5075	1.0569	0.0000	-0.0004	0.0001	0.0000
		out	CPUE	4.0998	0.8507	0.0000	0.0002	0.0001	0.0951
		in	WPUE	4.0120	1.5503	0.0097	0.0001	0.0001	0.2046
		out	WPUE	6.4912	1.2585	0.0000	-0.0002	0.0001	0.1680
Sparidae	on	in	CPUE	3.1268	3.4386	0.3632	-0.0003	0.0002	0.1826
		out	CPUE	0.4512	1.9803	0.8198	0.0002	0.0003	0.4371
		in	WPUE	0.9551	6.3711	0.8808	0.0002	0.0005	0.6705
		out	WPUE	15.0406	3.8232	0.0001	-0.0010	0.0005	0.0731
	off	in	CPUE	-0.0424	1.6429	0.9794	-0.0000	0.0001	0.6829
		out	CPUE	-1.6629	1.2989	0.2005	0.0001	0.0001	0.3791
		in	WPUE	2.4932	2.9286	0.3946	0.0002	0.0002	0.4174
		out	WPUE	-0.1526	2.3769	0.9488	0.0002	0.0003	0.5195
Serranidae	on	in	CPUE	-8.4988	5.9887	0.1559	0.0004	0.0004	0.3839
		out	CPUE	4.2759	3.3560	0.2026	-0.0008	0.0005	0.0887
		in	WPUE	0.1228	10.4832	0.9907	0.0000	0.0008	0.9531
		out	WPUE	4.5061	6.3008	0.4745	-0.0002	0.0009	0.7787
	off	in	CPUE	8.2760	1.8378	0.0000	-0.0006	0.0001	0.0000
		out	CPUE	6.6183	1.5054	0.0000	0.0001	0.0002	0.6702
		in	WPUE	11.2051	2.8104	0.0001	-0.0004	0.0002	0.0252
		out	WPUE	9.8436	2.2608	0.0000	0.0000	0.0003	0.8993
Labridae	on	in	CPUE	3.1268	3.4386	0.3632	-0.0003	0.0003	0.1826
		out	CPUE	0.4512	1.9803	0.8198	0.0002	0.0003	0.4371
		in	WPUE	0.9551	6.3711	0.8808	0.0002	0.0005	0.6705
		out	WPUE	15.0406	3.8232	0.0001	-0.0010	0.0005	0.0731
	off	in	CPUE	-0.0424	1.6429	0.9794	-0.0000	0.0001	0.6829
		out	CPUE	-1.6629	1.2989	0.2004	0.0001	0.0001	0.3791
		in	WPUE	2.4932	2.9286	0.3946	0.0002	0.0002	0.4174
		out	WPUE	-0.1526	2.3769	0.9488	0.0002	0.0003	0.5195
