# Supplementary material for: Marine reserve benefits and recreational fishing yields: The winners and the losers
Source: PLoS One. 2020 Dec 10;15(12):e0237685. doi: 10.1371/journal.pone.0237685 (PMC7728224; doi:10.1371/journal.pone.0237685)

S1 Fig Trends in catch per unit effort (CPUE) and weight per unit effort (WPUE) of recreational fishermen performing inside (red) vs outside (purple) the Cerbère-Banyuls marine reserve expressed in different ways: mean trajectory ±95% confidence interval with unit effort in line.hours (a and b), mean trajectory and observed data points with unit effort in line.hours (c and d), and mean trajectory and observed data points with unit effort in hook.hours (e and f). Trajectories were calculated using generalized linear models that accounted for a negative-binomial distribution of data points around the mean curves. Plots c and d show the same curves as in a and b, respectively (note differences in scale). For clarity, the format using mean trajectory ±95% confidence interval, rather than showing raw data points, is presented in manuscript. Similar results were found when considering fishing effort in line.hours (a-d) or hook.hours (e and f).

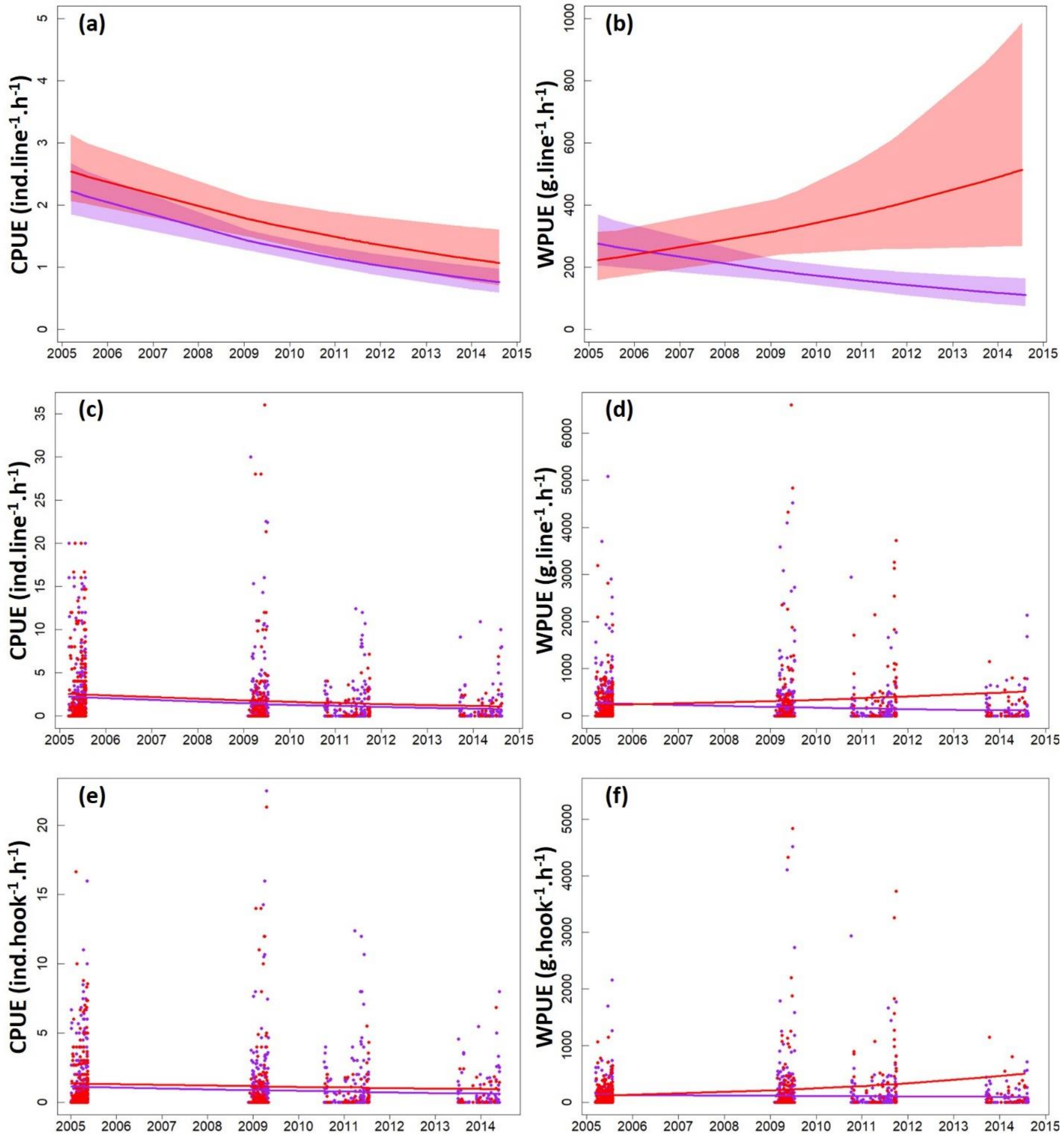

Supplement: S1 Fig — (PDF) [file pone.0237685.s006.pdf]
